# Supplementary material for: Active Huygens’ metasurface based on in-situ grown conductive polymer
Source: Nanophotonics. 2023 Dec 25;13(1):39–49. doi: 10.1515/nanoph-2023-0562 (PMC11502067; doi:10.1515/nanoph-2023-0562)
Supplement: Supplementary file 1 — Supplementary Material Details [file j_nanoph-2023-0562_suppl_001.docx]

**Supporting Information for**

**Active Huygens’ metasurface based on in-situ grown conductive polymer**

***Wenzheng Lu*, Leonardo de S. Menezes, Andreas Tittl, Haoran Ren and Stefan A. Maier****

**Table of contents**

Figure S1. Refractive index of polyaniline

Figure S2. Simulation of electrically active Huygens’ nanoantennas

Figure S3. Simulated electric field distribution

Figure S4. Optical measurement setup

Figure S5. Cyclic voltammogram for electrochemical polymer growth

Supplementary Note 1. In-situ polymer electrochemical growth on dielectric metasurfaces

Figure S6. Thickness measurement of PANI layer

Figure S7. SEM image of PANI-coated metasurface.

Figure S8. Transmitted energy contrast

Figure S9. Hysteresis behavior of electrical switching

Movie S1. Real-time electrical switching of the active Huygens’ metasurface

References

**
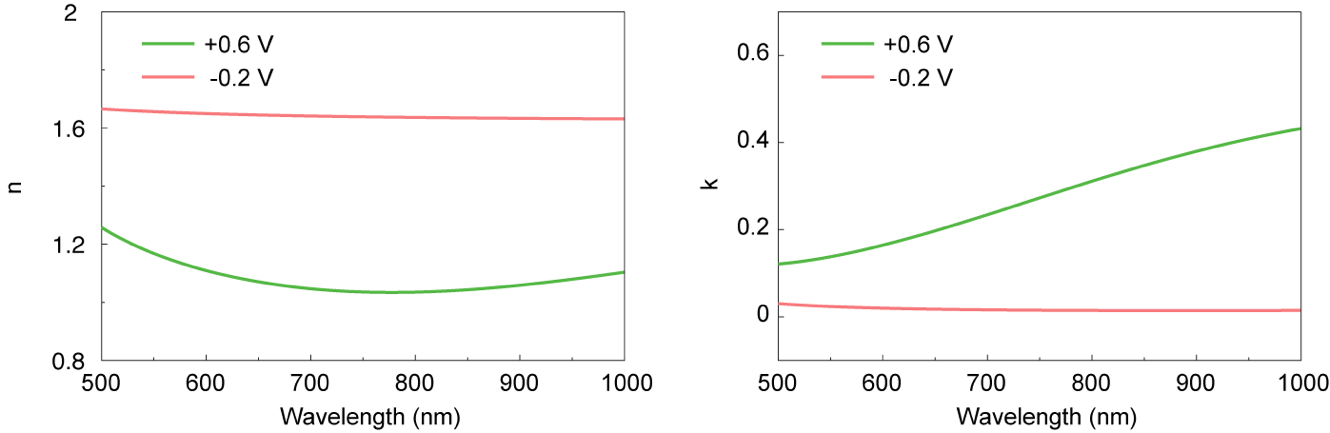
**

**Figure S1:** Refractive index of polyaniline. Real part (left) and imaginary part (k) of refractive index of polyaniline (PANI) in the oxidized state (+0.6 V, green line) and the reduced state (-0.2 V, red line), respectively. The refractive index was experimentally measured by an ellipsometer on a 100-nm thick electrochemically prepared PANI film on an indium-tin-oxide (ITO)-coated fused silica substrate.

**
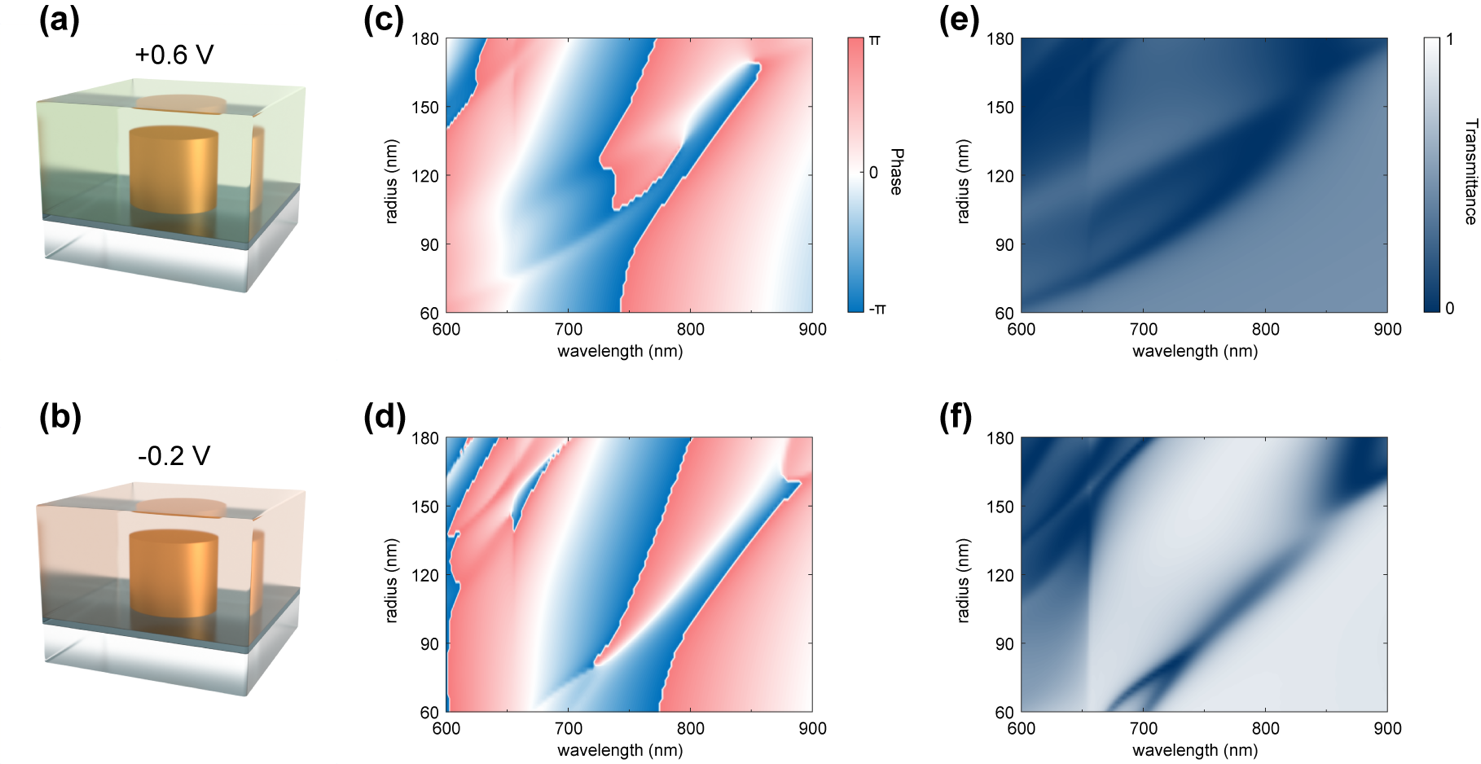
**

**Figure S2:** Simulation of electrically active Huygens’ nanoantennas. (a–b) Schematics of an individual nanoantenna made of a silicon nanodisk surrounded by a layer of PANI at an applied voltage of +0.6 V and -0.2 V, respectively. (c–d) Simulated transmission optical phase profiles (color-coded) of the nanoantenna at +0.6 V and -0.2 V, respectively. (e–f) Simulated transmittance (color-coded) of the nanoantenna at +0.6 V and -0.2 V, respectively.


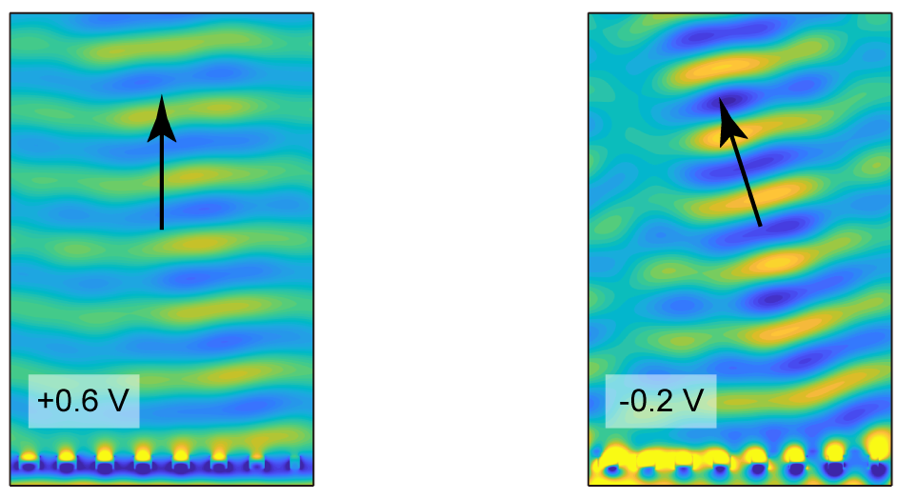


**Figure S3:** Simulated electric field distribution. Simulated electric field distribution of the transmitted light for the electrically active metasurface under a normal incident at an applied voltage of +0.6 V (left) and -0.2 V (right), respectively. The arrow indicates the k vector of the transmitted light propagating wave.

**
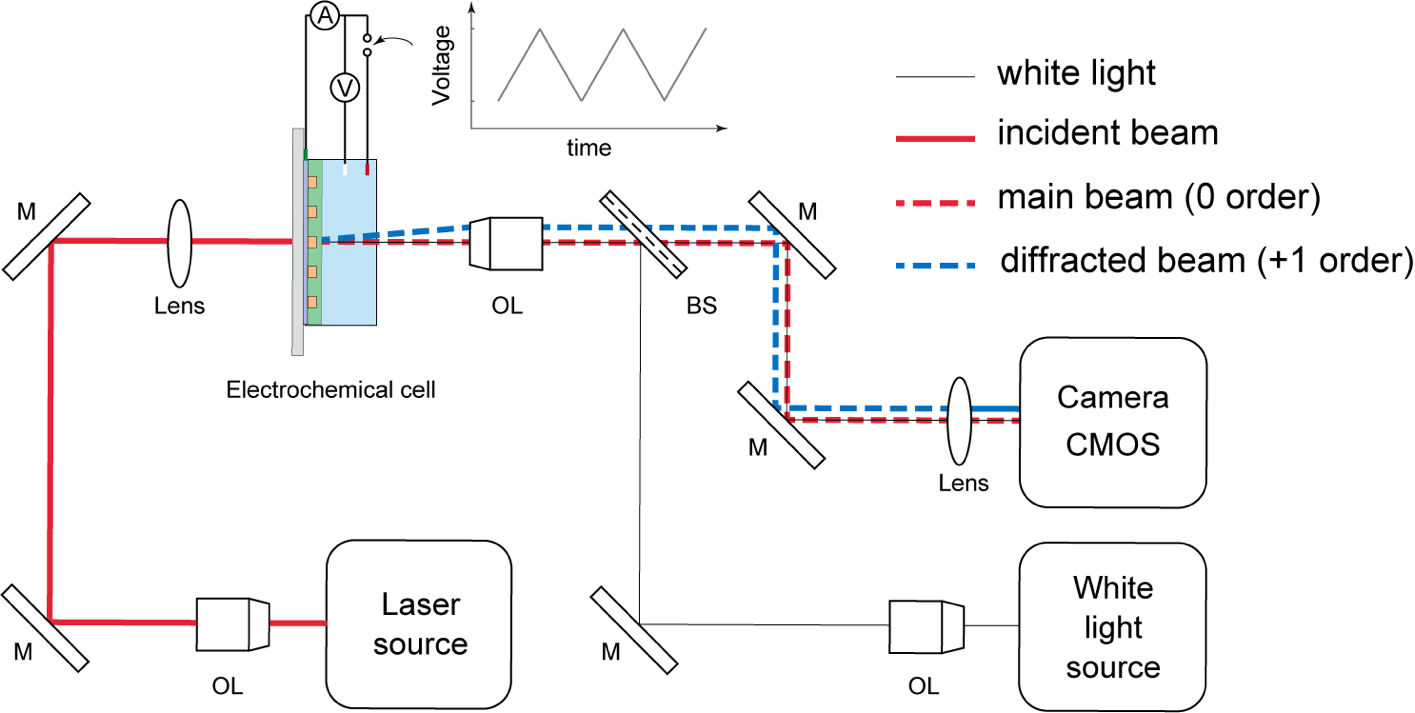
**

**Figure S4:** Optical measurement setup. Schematic of the optical measurement setup integrated with the custom-built electrochemical cell for measuring the transmitted intensities of different diffraction orders. The white light source is used for locating the metasurface sample, and is turned off when performing optical measurement. During the polymer growth and the electrical switching, a 785-nm laser is used to illuminate at the center of the metasurface, while a cycling voltage is applied on the metasurface to induce variation of transmitted diffraction pattern. The transmitted diffraction pattern is recorded by a high-speed monochromatic camera CMOS. OL: objective lens, BS: beam splitter, M: plane mirror.

**
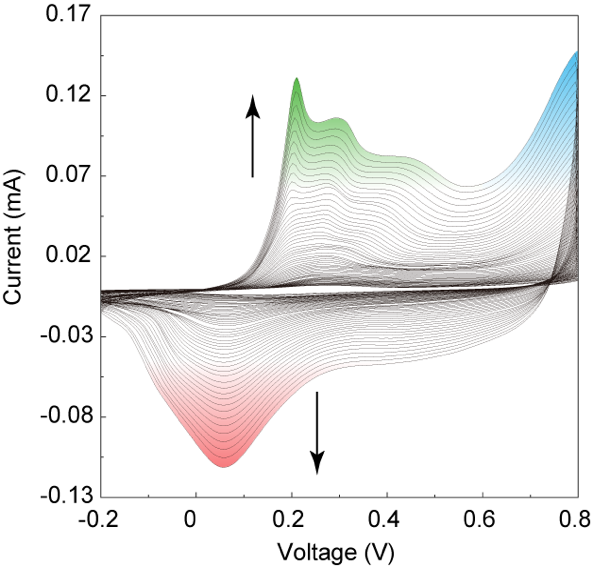
**

**Figure S5:** Cyclic voltammogram for electrochemical polymer growth. The measured cyclic voltammogram for the electrochemical growth of PANI on the dielectric metasurface, including 60 coating cycles. The green area shows the region where the PANI oxidation process takes place, whereas the red area shows the reduction process. The blue area shows the oxidative polymerization process of aniline, resulting in an increasing PANI thickness (see also supplementary note 1). The arrow indicates the increasing current intensity as more PANI is grown on the metasurface.

**Supplementary Note 1.** In-situ electrochemical polymer growth on dielectric metasurfaces. In the presence of the precursor monomer, in this case, aniline, in the electrolyte, application of a high voltage can trigger an oxidative polymerization on the surface of the ITO substrate directly from the electrolyte^1^. The dielectric metasurfaces resting on the top of the ITO substrate exposed to the electrolyte can thus be grown with PANI. The voltage for oxidative polymerization of aniline in our case is approximately +0.8 V (vs. Ag/AgCl reference), as depicted by the rising current intensity in the blue area of the cyclic voltammogram in Supplementary Figure 5. On the other hand, PANI can be switched between the oxidized state and the reduced state by an applied voltage in the range from +0.6 V to -0.2 V. Therefore, a cycling voltage in the range between -0.2 V and +0.8 V is able to induce polymer growth while switching the PANI after each increment in the PANI thickness, thus allowing for an in-situ optimization of PANI thickness on the beam steering performance. The oxidation and reduction processes of PANI are recorded by the oxidation peak and reduction peak on the cyclic voltammogram. The increasing amount of PANI grown on the metasurface results in an increasing oxidization current and reduction current, as displayed on the green and red area of cyclic voltammogram in Supplementary Figure 5.

**
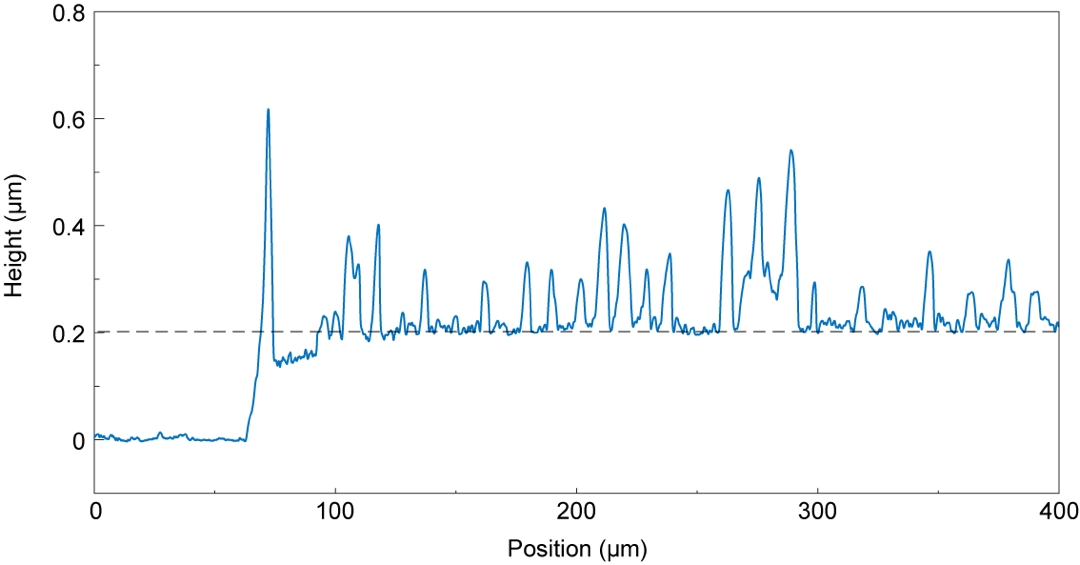
**

**Figure S6:** Measurement of PANI layer thickness. Thickness profile of the PANI layer grown on the metasurface. The PANI layer is optimized at a coating cycle number of 56, which has a thickness of approximately 200 nm. The thickness of the PANI layer is measured by a profilometer. The appeared spikes in the profile is caused by the polymer surface roughness.

**
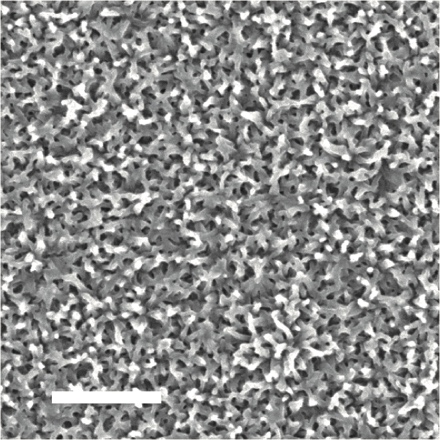
**

**Figure S7:** SEM image of PANI-coated metasurface. The PANI layer (thickness: 200 nm) has entirely covered the Si resonators (Hight: 140 nm) and shows fiber-like structure at nanometer scale. Scale bar: 1 μm.

**
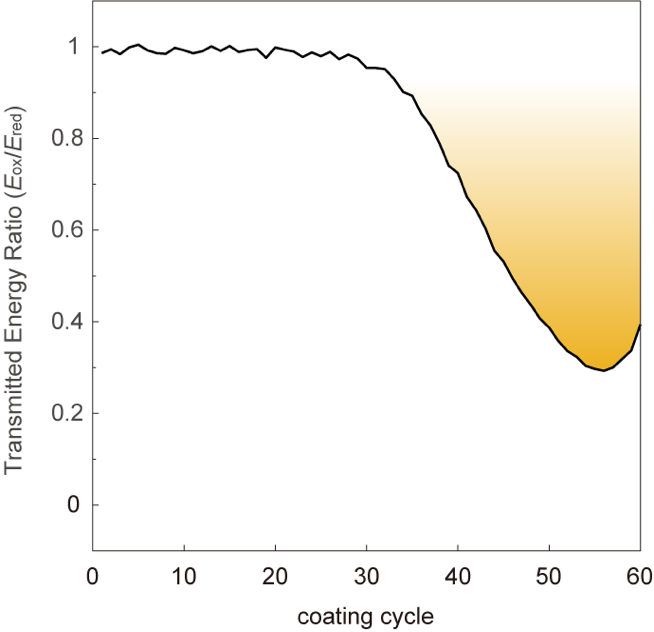
**

**Figure S8:** Transmitted energy contrast. Transmitted energy ratio between the oxidized state and the reduced state (*E*_ox_/*E*_red_) at different polymer coating cycles. The transmitted energy is calculated from the sum of the transmitted intensities of +1, 0 and -1 diffraction orders.

**
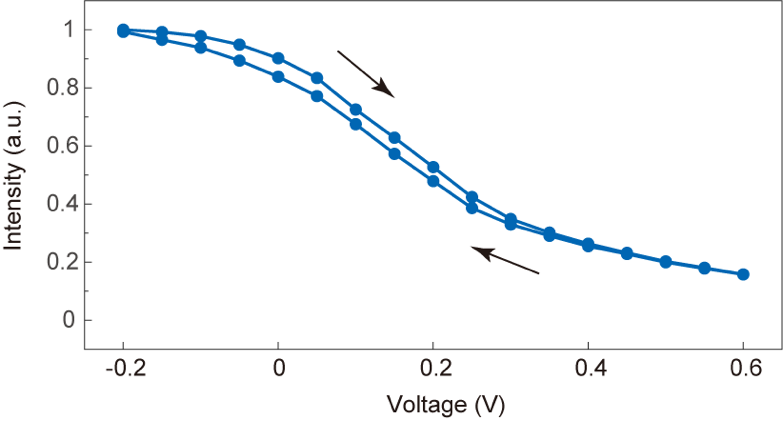
**

**Figure S9:** Hysteresis behavior of electrical switching. Diffraction intensity of the +1 order at different applied voltage switched from the oxidized state and the reduced state. The arrows indicate the electrical switching direction. The narrow gap between the intensity of the two switching directions shows that the electrical switching of the PANI-integrated metasurface is nearly hysteresis-free, allowing for the accurate electrical controllability over the intermediate states.

**Movie S1:** Real-time electrical switching of the active Huygens’ metasurface. Images of the transmitted diffraction pattern during the electrical switching for 9 complete cycles. The video including the real-time applied voltage (top left), the cyclic voltammogram (top right), the intensity of +1 diffraction order (bottom left) and the intensity of 0 diffraction order (bottom right). The slightly spike-like signal of the 0 order diffraction intensity is caused by the dynamic variation of absorption coefficient of PANI when the applied voltage reverses at around +0.6 V, which is observed in other PANI-based demonstration^2^. The video runs at 30 fps.

**References**

1. W. Lu, T. H. Chow, Y. Lu, and J. Wang, “Electrochemical coating of different conductive polymers on diverse plasmonic metal nanocrystals,” *Nanoscale*, vol. 12, no. 42, pp. 21617–21623, 2020.
2. R. Kaissner, J. Li, W. Lu, et al., “Electrochemically controlled metasurfaces with high-contrast switching at visible frequencies,” *Sci. Adv.*, vol. **7**, no. 19, p. eabd9450, 2021.
